# Subcellular compartmentalization of two calcium binding proteins, calretinin and calbindin-28 kDa, in ganglion and amacrine cells of the rat retina

**Published:** 2008-08-31

**Authors:** Deb Kumar Mojumder, Theodore G. Wensel, Laura J. Frishman

**Affiliations:** 1 Verna and Marrs McLean Department of Biochemistry and Molecular Biology, Baylor College of Medicine, Houston, TX; 2 University of Houston College of Optometry, Houston, TX

## Abstract

**Purpose:**

Intracellular free calcium ions (Ca^2+^) are an important element in retinal ganglion cell response. Two major EF-hand (E-helix-loop-F-helix-hand) calcium binding proteins in the retina, calretinin and calbindin-28 kDa, are important buffers of intracellular free Ca^2+^ in neurons, and may also serve as Ca^2+^-dependent regulators of enzymes and ion channels.

**Methods:**

This study used immunohistochemistry to investigate the subcellular expression patterns of calretinin and calbindin-28 kDa, in the soma, dendrites, and the axonal compartment of rat retinal ganglion cells.

**Results:**

Antibodies for calretinin and calbindin-28 kDa labeled different cell populations in the retinal ganglion cell layer. In this layer, calretinin labeled a larger number of cells compared to calbindin-28 kDa, many, but not all, of which were displaced amacrine cells. The calbindin-28 kDa immunopositive neurons were distinct in that their somata were peripherally encircled by microtubule associated protein 1 (MAP-1) or neurofilament-200 kDa subunit (NF-200 kDa) immunofluorescence. Although somata of retinal ganglion cells contained these calcium binding proteins, neither protein was found in the dendrites or initial segments of the axons. However, both were expressed in the ganglion cell axons in nerve fiber layer. Calretinin and calbindin-28 kDa staining overlapped in some fibers and not in others. Calretinin immunofluorescence was concentrated in discrete axonal regions, which showed limited staining for calbindin-28 kDa or for NF200 kDa, suggesting its close proximity to the plasma membrane.

**Conclusions:**

There is a clear compartmentalization of calbindin-28 kDa and calretinin distribution in retinal ganglion cells. This suggests that the two calcium binding proteins perform distinct functions in localized calcium signaling. It also indicates that rather than freely diffusing through the cytoplasm to attain a homogeneous distribution, calbindin-28 kDa and calretinin must be bound to cellular structures through interactions that are likely important for their functions.

## Introduction

Retinal ganglion cells (RGCs), the final output neurons of the retina, gather visual information from bipolar cells and amacrine cells by synaptic inputs from these neurons. They encode visual signals into Na+-dependent action-potentials that are transmitted along the optic nerve to higher visual centers in the brain. Both low-threshold and high-threshold Ca^2+^ channels present in RGCs contribute to their responses (for a review, see [1]). Indirectly, Ca^2+^ via Ca^2+^-activated K+ channels present in RGCs [2,3] can contribute to K+-dependent after-hyperpolarization following action potentials, which in turn can control excitability and firing patterns of neurons [4,5]. In the dendrites of RGCs, synaptic currents have been found to activate T-type calcium channels [6,7] which can augment and shape transient synaptic responses [8]. Changes in intracellular Ca^2+^ can also modulate ion channels, signaling cascades, and neurotransmitter receptors locally [2,9-17]. Impaired regulation of Ca^2+^ by calcium-binding proteins has been suggested to contribute to neurodegenerative processes [18,19], and changes in intracellular Ca^2+^ in RGCs have been proposed to play a role in excitatory neurotoxicity [20], inactivation of calpain [21] and other proteases, and in apoptotic cell death [22,23].

**Figure 1:**
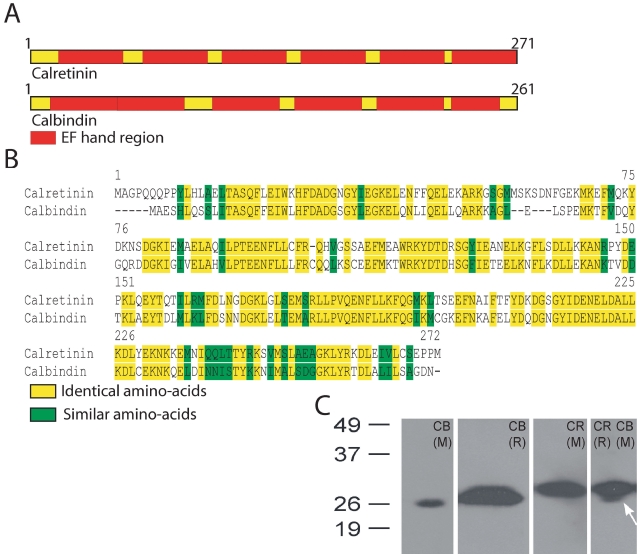
Schematic representation of calretinin and calbindin-28 kDa proteins and their sequence identity. **A:** Shown is a schematic representation of calretinin and calbindin-28 kDa proteins. The red blocks mark the E-helix-loop-F-helix-hand (EF) hand regions within each molecule. **B:** Alignment of the amino acid sequences of rat calretinin and calbindin-28 kDa molecules is based on NCBI accession numbers P47728 and P07171 respectively. Protein sequences were obtained from the NCBI protein database. **C:** Western blots for different calbindin-28 kDa (CB) and calretinin (CR) antibodies for rabbit (R) and mouse (M) are shown. Both calbindin-28 kDa and calretinin antibodies recognized a single protein band close to 26 kDa. The blot on the far right used antibodies for both calretinin (AB148) and calbindin-28 kDa (300). The arrow indicates the putative calbindin-28 kDa-positive band below the thicker calretinin positive band.

Changes in intracellular Ca^2+^ are modulated by calcium binding proteins (CBPs) that act as Ca2+ buffers, and these buffers are the major determinants of the kinetics of fluctuations in intracellular Ca^2+^ (for a review, see [24]). Calretinin and calbindin-28 kDa belong to a family of low molecular weight CBPs expressed in the retina and nervous system of vertebrates [25-30]. These proteins share approximately 59% sequence identity and 77% similarity (Figure 1B). Each has six E-helix-loop-F-helix-hand (EF)-hand motifs (Figure 1A), but only four are functional in calbindin-28 kDa and only five are active in calretinin [31,32].

Despite their similar amino-acid sequence, these two proteins are different in many respects. Structurally, they have disparate domain organizations of their EF-hand motifs [31], and functionally, they interact with different partners in various cells. For example, in calcium signaling pathways, calbindin-28 kDa interacts with caspase-3 [33] whereas calretinin interacts with cytoskeletal components [34] and basic helix–loop–helix transcription factors [35]. Under pathological conditions, such as in response to ischemia and reperfusion, their levels in RGCs are differentially altered [36]. Their distinctive functions are highlighted by their presence in distinct neuronal populations in the central nervous system (for example [27,37-39]), where they may serve unique roles.

The goal of the present study was to go beyond the previous studies that investigated the distribution of calretinin and calbindin-28 kDa in the rat retina [27,40] and to examine the cellular and subcellular distributions of these proteins in the ganglion cell layer. This study shows that calbindin-28 kDa and calretinin have distinct compartmentalization in RGCs. This suggests that structurally certain intracellular quantities of these two CBPS must be bound to cellular structures. Functionally these bound proteins could influence subcellular Ca^2+^ signaling and local Ca^2+^ dynamics.

## Methods

### Animals for immunohistochemical studies

Studies were performed using 16 1-1.5-year-old Brown Norway rats (*Rattus norvegicus*; Charles River Laboratories, Wilmington, MA) and two three-month-old Sprague-Dawley rats (*Rattus norvegicus*; Charles River Laboratories, Wilmington, MA). All animals were maintained on a 12 h:12 h light-dark cycle. Food and water were available ad libitum. Animals were light-adapted before euthanasia—in room light that was rod saturating, at least 1 scot cd.m2. The Brown Norway rats were initially anesthetized using an intramuscular injection of 86 mg/kg ketamine and 6.5 mg/kg xylazine (Both drugs were from Vedco, St. Joseph, MO) and then euthanized by an intramuscular injection of 150–200 mg/kg pentobarbital. The two Sprague-Dawley rats were used for immunoblotting, and were euthanized by CO2 inhalation. All animal procedures and protocols conformed to the United States Public Health Service and Institute for Laboratory Animal Research guidelines and were approved by both the University of Houston Institutional Animal Care and Use Committee as well as the Baylor College of Medicine Institutional Animal Care and Use Committee.

### Tissue preparation for immunohistochemistry

Following euthanasia, the eyes were rapidly excised from the orbit. A portion of the superior rectus muscle was left to indicate the superior pole of the globe. The corneas were slit with a razor blade, the lens was removed, and eyes were immersed in 4% paraformaldehyde in 0.1 M sodium cacodylate buffer (pH 7.4) for 1 h at 4 °C. Following fixation, eyes were rinsed in phosphate buffered saline (PBS; pH 7.4) and cryoprotected in 30% sucrose in 1X PBS overnight. The next day, the eyes were embedded in Tissue-Tek OCT Compound (Tissue-Tek, Hatfield, PA) and fast frozen in liquid nitrogen. Vertical cryostat sections of 10–12 µm thickness were cut parallel to a plane tangent to the corneal surface at the center of the pupil and collected onto Superfrost/Plus microscope slides (Fisherbrand; Fisher Scientific, Pittsburgh, PA). Sections were stored at −20 °C until use.

For retinal whole-mounts, the eye was excised and the cornea and lens were removed as described in the previous section. Following removal of the sclera and the retinal pigmented epithelium, the neural retina along with some overlying vitreous was rapidly removed. The retina was rinsed in cold Ames’ medium (Sigma-Aldrich, St. Louis, MO; 4 °C, pH 7.4) and then immediately fixed in 4% paraformaldehyde in 0.1 M cacodylate buffer (pH 7.4) for 5 min at 4 °C. The vitreous humor was removed, and relaxing cuts were made in the retinal margin to allow the retina to flatten. The retina was rinsed in 1X PBS and subsequently incubated with the antibodies diluted to their appropriate concentration (Table 1). The details of the immunolabeling procedure has been described in detail below.

**Table 1:** Primary antibodies and antisera

**Antigen**	**Host**	**Dilution**	**Source**	**Reference**
Calbindin-28kDa	Mouse	1:1000-1:5000	SWANT, Bellinzona, Switzerland (Cat# 300)	[37], [27]
Calbindin-28kDa	Rabbit	1:1000-1:5000	SWANT, Bellinzona, Switzerland (Cat# CB38)	[27]
Calretinin	Rabbit	1:1000-1:5000	Chemicon International, Temecula, CA (Cat# AB148)	[60], [27]
Microtubule Associated Protein-1 (MAP-1)	Mouse	1:300	Sigma-Aldrich, St. Louis, MO (Cat# M4278; Clone HM-1)	[58], [59]
Neurofilament-200 kDa	Mouse	1:1000	Chemicon International, Temecula, CA (Cat# MAB5266; Clone N52)	[52], [59]
Na_V_ α-subunit, pan-specific (Pan-Na_V_)	Mouse	1:500-1:1000	Sigma-Aldrich, St. Louis, MO (Cat# S8809; Clone K58/35)	[49]
Na_V_1.1 α-subunit	Mouse	1:500-1:1000	G. Matthews (SUNY-Stony Brook), J. Trimmer (UC Davis), Antibodies, Inc./NeuroMab, Davis, CA (Cat# 75-023; Clone K74/71)	[50]
Na_V_1.2 α-subunit	Mouse	1:500-1:1000	J. Trimmer (UC Davis); Antibodies, Inc./NeuroMab, Davis, CA (Cat# 75-024; Clone K69/3)	[51]
Na_V_1.6 α-subunit	Mouse	1:500-1:1000	J. Trimmer (UC Davis), Antibodies, Inc./NeuroMab, Davis, CA (Cat# 75-026; Clone K87A/10)	[48]

Specificity, host, dilution, and source information for the primary antibodies and antisera that were used in these studies.

### Antibodies and antisera

The antibodies and antisera used are detailed in Table 1. Binding of primary antibodies was detected using fluorescent antisera. The secondary antisera used were raised in goat and specific for either mouse or sheep immunoglobulins and were conjugated to a 1:200 dilution for AlexaFluor488 or AlexaFluor546 (Invitrogen Corporation, Carlsbad, CA).

### Immunoblotting

Antibody specificity was tested using immunoblots of membrane homogenates. After the Sprague Dawley rats were euthanized by CO2 inhalation, one retina from each rat was rapidly extracted, rapidly frozen in dry ice, and powdered with a pestle. Added to the retina was a homogenization buffer composed of the following: 20 mM HEPES, pH 7.0, 150 mM NaCl, 3 mM MgCl_2_, 1 mM CaCl_2_, 1 mM beta-mercaptoethanol, 0.01% NaN_3_, 1 mM EDTA, 1X protease inhibitors, and solid phenylmethylsulphonyl fluoride (PMSF). The tissue was sonicated and then centrifuged at 12,000x g for 10 min. Protein concentration of the retinal homogenate was quantified using Bradford assay against a known BSA standard [41]. Supernatant containing approximately 70 µg of protein were loaded into each well of 12% SDS polyacrylamide gel. Molecular weight standards (BenchMark™ Protein Ladder; Invitrogen) were run on adjacent lanes. The gels were run at constant current to separate proteins. Proteins were transferred to nitrocellulose membranes and blocked with 5% fat-free milk in Tris-buffered saline with Tween-20 (TBST) buffer with 0.02% NaN3. Each membrane was then incubated with primary antibody, which had been diluted in 5% fat-free milk in TBST with 0.02% NaN3. A 1:1,000 dilution was used for all antibodies. The nitrocellulose membrane was rinsed and incubated in secondary antibody conjugated to 1:10,000 HRP. Protein bands were visualized by enhanced chemiluminescence.

### Immunolabeling

Immunofluorescent methods used in this study for immunolabeling frozen sections and retinal whole-mounts are described previously [42-45]. Frozen sections were thawed, rinsed in deionized water, treated with 1%–2% NaBH4 to reduce autofluorescence, rinsed in deionized water, followed by 1X PBS. Nonspecific labeling was attenuated with 10% normal goat serum, 5% BSA, 0.5%–1% fish gelatin,and 0.1%–0.5% Triton X-100 in PBS (“blocker”).

After removal of excess blocker, the primary antibody was incubated for 24–48 h at 4 °C in blocker. A combination of primary antibodies was applied simultaneously for double labeling experiments. Subsequently, sections were rinsed with 1X PBS, blocked for 30 min at room temperature, and secondary antibody was applied for 1 h at room temperature in blocker. An appropriate combination of secondary antisera was applied simultaneously for double labeling experiments. Sections were rinsed and coverslipped in a fade-retardant mounting medium (Vectashield; Vector Labs or Prolong Gold; Invitrogen) and examined with the microscope. As anticipated, there was no labeling in sections processed substituting normal rabbit serum for rabbit polyclonal primary antisera, nonspecific mouse IgGs, or in the absence of primary antibodies.

Rat retinal whole-mounts were immunolabeled free-floating (i.e., were incubated with the appropriate antibodies diluted in “blocker” in an eppendorpf tube, such that they were capable of free movement within the tube). Whole-mounts were treated with 1%–2% NaBH4 for 1–2 min, rinsed in deionized water followed by 1X PBS and incubated in blocker solution for 1 h at room temperature to block nonspecific labeling. Retinas were incubated in primary antibody for 5 days at 4 °C. Retinas were rinsed in 1X PBS for 2 h at room temperature and then incubated free-floating in secondary antibody at room temperature for 1–2 h. Retinas were then rinsed in 1X PBS for 2 h at room temperature, flattened onto microscope slides with the ganglion cell side up, coverslipped with a fade-retardant mounting medium (Prolong Gold; Invitrogen) and examined in the confocal microscope. For each antibody a minimum of three retinas from three different animals were tested. The results of this study were consistent for the antibody concentrations (Table 1) and detergent concentrations of 0.1 to 0.5% Triton X-100 used.

### Imaging

Images were acquired using a Leica TCS SP2 confocal microscope and LCS software (Leica Microsystems, Exton, PA). Images were captured using 20x (NA, 0.7), 63x oil (NA, 1.32), or 63x water immersion (NA, 1.2) objective lenses. Stacks of serial optical sections spaced from 1.5 µm to 6 µm apart in the Z plane were collected. For assessment of labeling in single optical planes, we used 63x objectives to achieve a maximal Z-plane resolution. Images in each fluorescent channel were collected sequentially with laser power and detector sensitivity adjusted to prevent bleed-through of signals between fluorescence channels. The absence of bleed-through between channels was confirmed in sections treated with a single primary antibody and a combination of secondary antibodies imaged using identical settings to verify that only the channel corresponding to the primary antibody showed labeling.

Figures were prepared by importing images into Adobe Photoshop 6.0 (Adobe Systems, Inc., Mountain View, CA) and calibrating image scale. To estimate colocalization of immunofluorescence from two different antibodies in the nerve fiber layer, we plotted the channel intensity of each label along its long axis by using the ImageJ software (W.S. Rasband, NIH, Bethesda, MD) and its red-green-blue (RGB)_Profiler plugin (Laummonerie and Mutterer, Institut de Biologie Moléculaire des Plantes, Strasbourg, France). To estimate cell soma size in the RGC layer, the outer border of the cell membrane, stained for Na_V_, was traced manually from confocal projection of the RGC layer. The area was then measured using ImageJ software (W.S. Rasband, NIH, Bethesda, MD).

## Results

### Immunoblotting

Western blots using the different primary antibodies against calbindin-28 kDa (300 and CB-38a) and calretinin (AB148) each labeled a single protein-band close to the molecular weight marker band of 26 kDa (Figure 1C), indicating that the antibodies were specific for proteins of that molecular weight. The mouse monoclonal antibody for calbindin-28 kDa, 300, (Figure 1C) used together with the rabbit polyclonal antibody for calretinin, AB148, revealed that the two antibodies recognized specific proteins that had different molecular weights. The single protein band labeled by the calbindin-28 kDa rabbit polyclonal antibody (CB-38a) was too broad to rule out cross-reactivity with calretinin.

### Labeling for calretinin and calbindin-28 kDa

**Figure 2:**
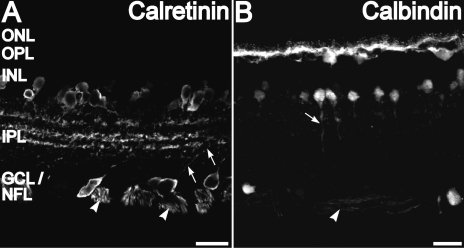
Calretinin and calbindin-28 kDa immunolabeling of a vertical cryosection. **A:** Calretinin immunolabeling was present in cell bodies and processes of amacrine cells at the inner nuclear layer (INL)-inner plexiform layer (IPL) border. Calretinin labeling was also present in cell bodies and processes (arrows) in the ganglion cell layer (GCL). Calretinin labeling is also found in three distinct bands in the IPL and retinal ganglion cell (RGC) axons in the nerve fiber layer (NFL; arrowhead). **B:** Calbindin-28 kDa immunolabeling was present in cell bodies and processes of horizontal cells at the outer plexiform layer (OPL)-INL border. Calbindin-28 kDa also labeled amacrine cells at the INL-IPL border. Some descending processes were seen for some of these neurons (arrow). There are also diffuse calbindin-28 kDa-positive punctate in the IPL. Calbindin-28 kDa labeling is seen in few cells in the GCL as well as in the RGC axons in the NFL (arrowhead). Scale bars represent 20 µm.

Staining for calretinin and calbindin-28 kDa in vertical sections of the retina was similar to previous results in rats (for example, see [40]). The somata and descending processes of some amacrine cells whose somata resided in the proximal inner nuclear layer (INL) close to the inner plexiform layer (IPL) were brightly stained by calretinin (Figure 2A). A large number of cell bodies in the RGC layer (GCL) were also stained with calretinin antibodies. The calretinin immunopositive cell bodies in the GCL are known to include the displaced amacrine cells in the rat retina [46]. Dendrites from some of the calretinin-positive neurons in the INL and GCL projected toward the narrow calretinin-positive bands in the IPL (arrows in Figure 2A). These calretinin immunopositive dendrites were found to originate from the displaced amacrine cells in the ganglion cell layer in the rat retina [46]. The nerve fiber layer (NFL), where the unmyelinated axons of the RGCs are located, was also immunoreactive for calretinin.

**Figure 3:**
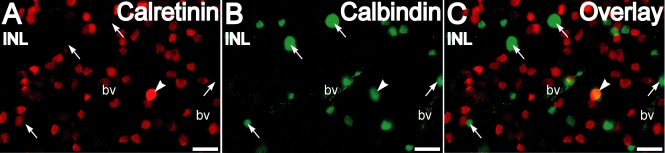
Calretinin and calbindin-28 kDa are differentially distributed in amacrine cells as seen in retinal whole-mounts. **A-C:** Double labeling for calretinin (red) and calbindin-28 kDa (green) in the inner nuclear layer shows that labeling for each was present in a distinct set of amacrine cells For the apparent region of overlap (arrowhead) in the overlay of this confocal plane, examination of different z-planes revealed that these were disparate cells located at different depths. Arrows indicate calbindin-28 kDa-immunopositive cells. Scale bar represents 20 µm. Abbreviations: bv is blood vessel.

Intense calbindin-28 kDa labeling was found in horizontal cells and their processes in the outer plexiform layer- inner nuclear layer (OPL-INL) border (Figure 2B) as observed in previous studies [27]. There was no significant staining for calbindin-28 kDa in the mid-INL where the cell bodies of the bipolar cells are located. Amacrine cells at the INL-IPL border showed staining for calbindin-28 kDa. Many of these cells had a single, stout apical process descending into the IPL. These cells did not have any calretinin labeling (see next section). In the IPL, the mouse monoclonal calbindin-28 kDa antibody showed punctate labeling. Calbindin-28 kDa labeling of somata in GCL with the mouse monoclonal antibody was sparse but axons in the NFL were labeled. On double-stained whole-mounts, calretinin and calbindin-28 kDa antibodies labeled different subsets of amacrine cells in the proximal INL (Figure 3) with almost no overlap, indicating that each antibody identified a specific amacrine cell type.

### Differential distribution of calretinin and calbindin-28 kDa in GCL/NFL

**Figure 4:**
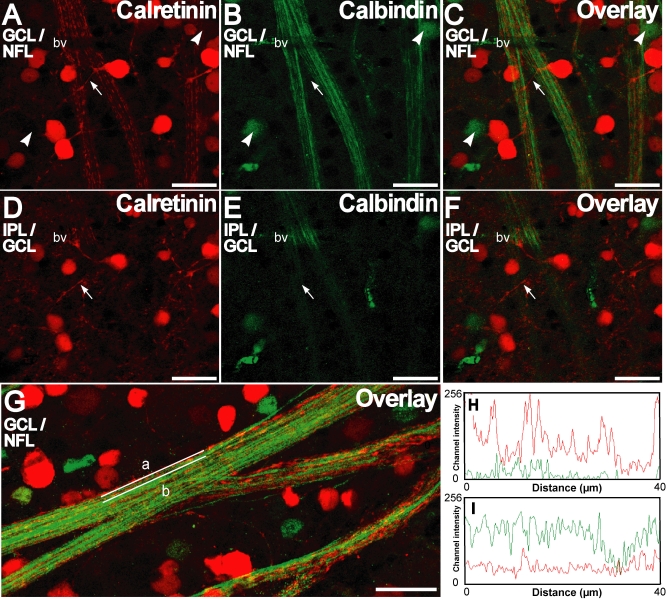
Calretinin and calbindin-28 kDa are distinctly distributed in the ganglion cell and nerve fiber layer as seen in retinal whole-mounts. **A-C:** Double labeling for calretinin (red) and calbindin-28 kDa (green) in the ganglion cell layer (GCL)/nerve fiber layer (NFL) shows that labeling for each was present in a distinct set of neurons. Calbindin-28 kDa positive cell bodies are indicated by arrowheads. Some calretinin-positive neurons show processes (arrow) that ascend distally. Note the discontinuous staining pattern of calretinin in the NFL in contrast to a smoother staining pattern for calbindin-28 kDa. **D**,**E:** A single confocal optical section distal to that of **A-C** shows that the calretinin positive processes (arrow) are directed in the inner plexiform layer (IPL) distally toward a calretinin-immunopositive plexus, a characteristic of displaced amacrine cells. **G:** Representative calretinin and calbindin-28 kDa double staining in the GCL/NFL is shown. Channel intensity profiles for the red and green channels for straight lines along the long axis (lines a and b in **G** shown in **H** and **I** respectively) show different intensity profiles for calretinin and calbindin-28kDa immunofluorescence. Scale bar represents 20 µm.

Double labeling immunofluorescence with mouse monoclonal anticalbindin-28 kDa and rabbit anticalretinin in whole-mounts showed that the two calcium binding proteins were present in the somata of random sets of the neurons in the GCL (Figure 4). In the whole-mounts, processes emanating from some of the calretinin immunopositive cells in the GCL were also stained. When followed through multiple optical sections, it was apparent that these processes were directed away from the GCL and NFL, and toward the IPL, where they merged with the proximal calretinin positive plexus in the IPL (Figure 4 D-F) as was observed in the vertical sections (see Figure 2A). Because this proximal calretinin positive plexus in the IPL also corresponds with the stratification of displaced amacrine cells it is evident that these dendrites originated from the displaced amacrine cells in the GCL and merged with the proximal cholinergic band in the IPL [46]. Calbindin-28 kDa immunopositive somata in the GCL were fewer in number and never showed any colocalization with calretinin. None of the somata staining for calbindin-28 kDa showed calbindin-28 kDa-immunopositive-processes emanating directly from the cell body

Calretinin and calbindin-28 kDa antibodies both stained the RGC axons in the NFL, but for the most part the staining patterns did not overlap. In the axonal compartment, calretinin antibodies showed punctuate staining at intermittent locations along the axons, on a background of diffuse immunofluorescence (Figure 4 A,G). The axons that were immunopositive for calbindin-28 kDa were fairly uniformly labeled along their length. The channel intensity profiles along the long axes of two nerve fiber bundles show that relative levels of staining for the two CBPs varied greatly from axon to axon, (Figure 4G-I), indicating that these two proteins are present in differing amounts in different axons. Sharp peaks in the calretinin intensity profile demonstrated that calretinin was concentrated at distinct locations in the axons unlike calbindin-28 kDa (Figure 4H).

### Dendritic compartments of retinal ganglion cells are devoid of immunostaining for calretinin and calbindin-28kDa

**Figure 5:**
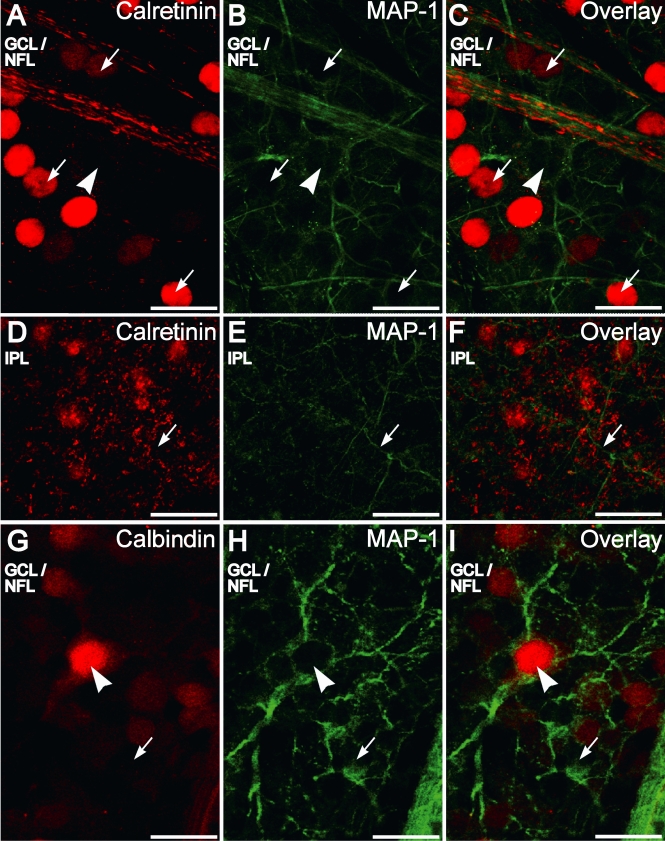
Calretinin and calbindin-28 kDa immunofluorescence is not present in the distal dendritic compartment of retinal ganglion cells as seen in retinal whole-mounts. **A-C:** Double labeling for calretinin (red) and microtubule-associated protein 1 (MAP-1; green) in the ganglion cell layer (GCL)/nerve fiber layer (NFL) shows that MAP-1 positive dendrites are not colabeled with calretinin. Some large retinal ganglion cells (RGCs) that are completely ringed by MAP-1 staining (arrowhead) are not positive for calretinin. RGCs with smaller somata partially ringed with MAP-1 staining are immunopositive for calretinin (arrows). Other brightly stained somata not showing MAP-1 immunofluorescence are the displaced amacrine cells that also stained for calretinin. **D-F:** Confocal plane showing that the MAP-1 positive (green) dendrites (arrow) do not merge with the calretinin-positive plexus (red) in the inner plexiform layer. **G-I:** Double labeling for calbindin-28 kDa (red) and MAP-1 (green) in the GCL/NFL shows that some large RGCs that are completely ringed by MAP1 staining (arrowhead) are positive for calbindin-28 kDa. Some RGCs with smaller somata that are incompletely ringed with MAP-1 are also calbindin-28 kDa positive while others are not (arrow). Some neurons incompletely ringed with MAP-1 are also not calbindin-28 kDa positive (arrow). Scale bar represents 20 µm.

Calbindin-28 kDa did not label any processes that ascended distally from the GCL. The processes labeled by calretinin that ascended distally from the GCL were likely from displaced amacrine cells in the GCL, as noted in the previous section.. To characterize further the morphology of the ganglion cells whose somata were stained for calretinin and calbindin-28 kDa, double labeling for microtubule associated protein 1 (MAP-1) and calretinin or calbindin-28 kDa was performed in whole-mounts. MAP-1 is known to label the dendrites of RGCs in rats [47]. In the IPL/GCL MAP-1 labeled the dendrites of RGCs (Figure 5) but for almost their entire length these did not stain for either CBP. Calbindin was found to be present at the very proximal portions of the RGCs but absent more distally (Figure 5G-I). These results indicate that neither calretinin nor calbindin-28 kDa was present in detectable levels in the dendritic compartment of the RGCs.

MAP-1 immunopositive dendrites did not stratify extensively with the calretinin-positive plexuses in the IPL (Figure 5D-F). However, calretinin-positive dendrites emanating from the displaced amacrine cells in the proximal INL and GCL did stratify in the calretinin-positive bands in IPL (Figure 4D-F).

**Figure 6:**
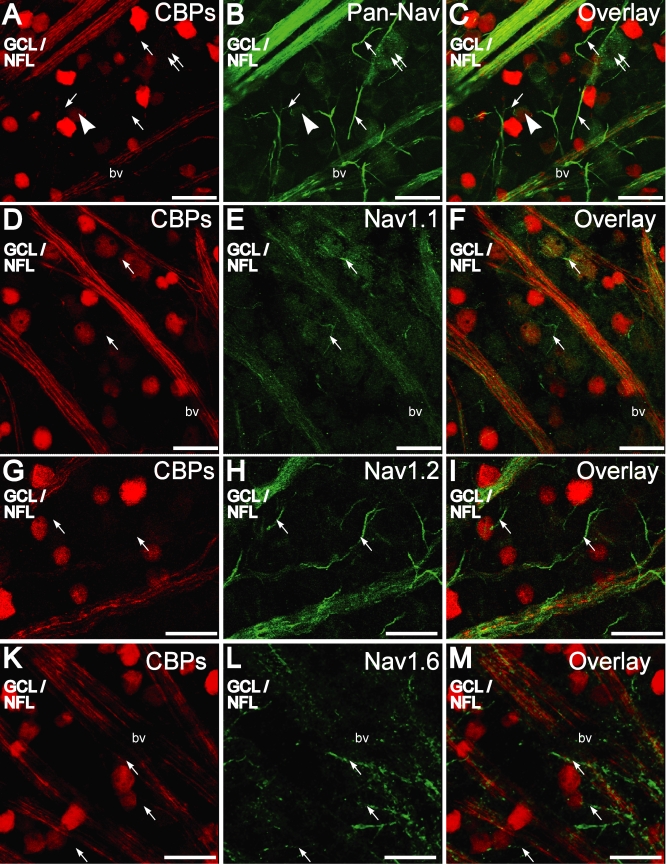
Comparison of immunofluorescence for the calcium binding proteins, calretinin and calbindin-28 kDa, and voltage-gated sodium channel antibodies in the ganglion cell layer (GCL)/nerve fiber layer (NFL) as seen in retinal whole-mounts. **A-C:** Calcium binding proteins (CBP) in the NFL are extensively colocalized with Pan-NaV. Some Pan-Na_V_ stained retinal ganglion cell (RGC) somata were also immunopositive for CBPs (arrowhead) while others were not (double arrows). Initial segments of RGCs, (arrow) some of which can be seen emerging from the RGC somata, were immunopositive for Pan-Na_V_ but not colabeled with CBPs. **D-F:** Na_V_1.1-immunopositive (green) RGC nerve fiber bundles in the nerve fiber layer (NFL) were colabeled with CBPs (red), but the axon initial segments (arrow) were not. **G-I:** Na_V_1.2 immunopositive (green) RGC nerve fiber bundles in the NFL were colabeled with CBPs (red) but not the axon initial segments (arrow). **J-K:** Na_V_1.6 immunopositive (green) axon initial segments (arrow) were not colabeled with CBPs (red). Scale bar equals 20 µm. Abbreviations: bv is blood vessel.

MAP-1 completely encircled the periphery of the cell body in some large RGCs (Figure 5A-C,G-I arrowhead). These large RGCs somata were not calretinin positive (Figure 6A-C), but some were calbindin-28 kDa immunopositive (Figure 5G-I). Yet, many smaller RGC somata that were incompletely encircled by MAP-1 immunofluorescence were found to be calretinin positive (arrows, Figure 5A-C) and calbindin-28 kDa negative (arrow, Figure 5G-I). Displaced amacrine cells in the GCL that were brightly stained for calretinin did not have MAP-1 staining in their dendritic processes.

### Immunofluorescence for calretinin and calbindin-28 kDa are absent in the RGC initial segments

RGC initial segments that emanate from the RGC somata show clustering of specific isoforms of voltage-gated sodium channels [48]. To label these initial segments an antibody specific for all Na_V_1 α-subunits (Pan-Na_V_) [49] and those for Na_V_1.1 [50], Na_V_1.2 [51], or Na_V_1.6 [48] α-subunits were used. Na_V_1.1 and Na_V_1.2 α-subunit antibodies are also known to label some processes in the IPL, whereas, Na_V_1.6 α-subunit antibody does not [50]. The initial segments in the GCL/NFL that were immunopositive for Pan-Na_V_, Na_V_1.1, Na_V_1.2, or Na_V_1.6 α-subunit antibodies did not show immunofluorescence for either calretinin or calbindin-28 kDa, indicating their absence in the initial segments of the RGCs. A combination of staining for calretinin and calbindin-28 kDa is seen in the red channel (Figure 6).

**Figure 7:**
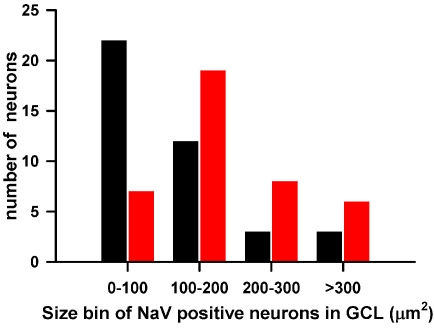
Size distributions of Calcium-binding protein (CBP) immunopositive and immunonegative neurons are shown. The histogram shows the surface area of projection of the somata of neurons in the retinal ganglion cell (RGC) layer from retinal whole mounts that were immunopositive for Pan-Na_V_ only (n=40, red) or Pan-Na_V_ and calcium binding proteins (CBP; n=40, black). The histogram is based on projections of all optical planes corresponding to the RGC layer from five midperipheral retinal areas (256 μm × 256 μm).

Some large neuronal somata were immunopositive for the Na_V_ α-subunits (for example, Figure 6A-C, double arrow); these did not show immunofluorescence for calretinin or calbindin-28 kDa. The Pan-Na_V_ antibody delineated the cell membrane, making it possible to trace the outer margin of the cell soma and calculate its surface area of projection in whole-mounts as an estimate of cell size. Of the cell counted, sizes varied for CBP immunopositive and CBP immunonegative neurons (Figure 7). The smaller sized somata most likely included displaced amacrine cells. In addition, 15% of CBP immunonegative neurons and 7.5% of CBP immunopositive neurons showed somatic sizes that were greater than 300 µm2. Based on somatic size both the CBP and non-CBP immunopositive cells represented a heterogeneous population of cells,

### Distribution of calretinin and calbindin-28 kDa relative to NF-200 kDa in the axonal compartment

**Figure 8:**
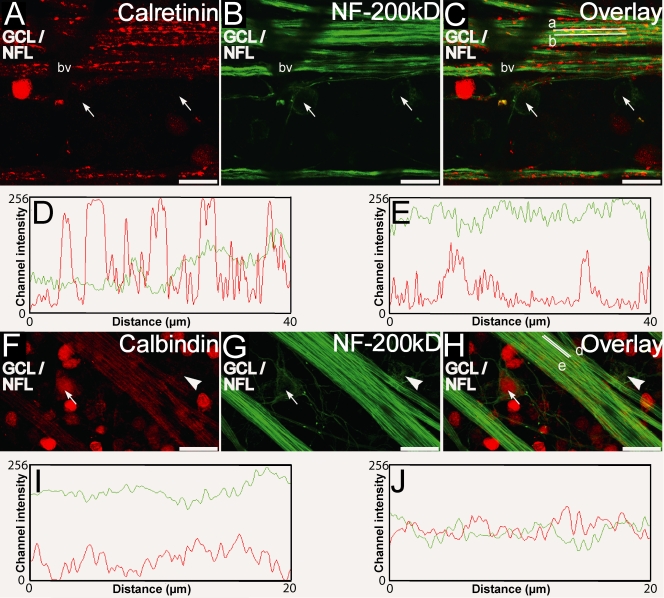
Calretinin, calbindin-28 kDa, and NF-200 kDa immunofluorescence in the ganglion cell layer (GCL)/nerve fiber layer (NFL) as seen in retinal whole-mounts. **A-C:** Double labeling for calretinin (red) and NF-200 kDa (green) shows retinal ganglion cell (RGC) somata, surrounded peripherally by light NF-200 kDa immunofluorescence (arrows), that were not immunopositive for calretinin. Calretinin immunofluorescence was present at discrete locations intermittently along the long axis of the RGC nerve fiber, whereas the NF-200 kDa immunofluorescence was uniform. Channel intensity profiles for the red and green channels along the long axis (lines a and b in **C** shown in **D** and **E** respectively) revealed that for regions on the long axis where staining for calretinin was prominent, staining for NF-200 kDa was less prominent (**D**) and vice versa (**E**). **F-H:** Double labeling for calbindin-28 kDa (red) and NF-200 kDa (green) showed that calbindin-28 kDa-positive immunofluorescence was smoothly distributed in the nerve fibers similar to NF-200 kDa. RGC somata that were surrounded peripherally by light NF-200 kDa immunofluorescence (arrow) were also stained with calbindin-28 kDa while for others (arrowhead) staining was less prominent. Channel intensity profiles for the red and green channels for straight lines along the long axis (lines d and e in **H** shown in **I** and J respectively) presented some region where immunofluorescence for NF-200 kDa was prominent while that for calbindin-28 kDa was less prominent (**I**) and others where the intensity profiles were similar (**J**). Scale bar represents 20 µm.Abbreviations: bv is blood vessel.

NF-200 kDa [52] was used together with the CBP antibodies to label the axons of the RGCs. In Figure 8A-C, the discrete regions in the axons where calretinin was concentrated showed limited immunofluorescence for NF 200 kDa, indicating different subcellular localization for these proteins. In contrast, patterns of calbindin-28 kDa immunofluorescence colocalized with NF-200 kDa in several locations (Figure 8J), but showed limited colocalization in other areas (Figure 8I). This demonstrates that calbindin-28 kDa was present in significant quantities in many but not all axons, where they were present in close proximity to neurofilaments in the cytoplasm. No CBP was visible in portions of axons immediately juxtaposed to the cell body, again indicating their absence in the initial segments of the axons. These results show that the differential distribution of calretinin and calbindin-28 kDa, noted in Figure 4G, could be because of their different subcellular locations in the RGC axons. High concentrations of calbindin-28 kDa together with lower diffuse concentrations of calretinin were most likely present in the cytoplasmic core in close proximity to NF-200 kDa, whereas high concentrations of calretinin were most likely present at discrete locations on the RGC membrane.

Similar to MAP-1 immunofluorescence, NF-200 kDa immunofluorescence was detected circumferentially around the somata of some ganglion cells (Figure 8B,G). The somata of these ganglion cells showed scant calretinin immunofluorescence; however, some (arrow, Figure 8F-H), but not others (arrowhead, Figure 8F-H), expressed calbindin-28 kDa, indicating another morphological difference between the RGCs that expressed calbindin-28 kDa and those that expressed calretinin. Along the length of axons in the NFL, relatively uniform neurofilament staining was largely coincident with calbindin-28 kDa staining but contrasted with the very punctate calretinin staining.

### Antibody specificity

Although some cross reactivity of the calbindin-28 kDa rabbit polyclonal antibody (CB-38a) with calretinin cannot be ruled out by the immunoblotting results, our interpretation that calbindin-28 kDa labeled a specific subset of ganglion cells is straightforward; because the calretinin antibody did not show cross-reactivity for calbindin-28kDa as revealed by its failure to label the calbindin-28 kDa immunopositive horizontal cells or the calbindin-28 kDa-immunopositive amacrine cells (Figure 3). In addition, the calbindin-28 kDa-immunolabeled RGCs have morphology clearly distinct from those staining with calretinin, as seen by colabeling with MAP-1 and NF-200 kDa antibodies. Thus, under the conditions used for immunostaining, the antibodies appear to be highly specific, and clearly reveal distinct staining patterns in the RGC axons.

## Discussion

**Figure 9:**
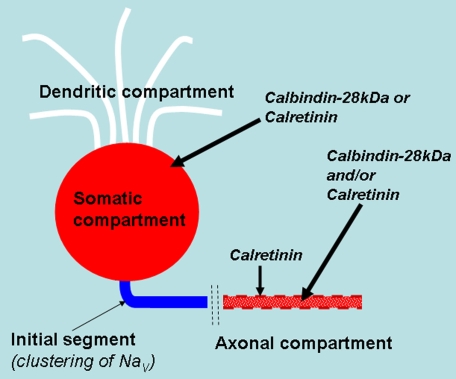
Schematic summarizing the subcellular distribution of calbindin-28 kDa and calretinin in the different compartments of the retinal ganglion cell. Labeling for calbindin-28 kDa or calretinin was absent from the dendritic compartment of the retinal ganglion cells (white). Many, but not all, retinal ganglion cell soma show labeling for either calbindin-28 kDa or calretinin (red). The initial segments of the retinal ganglion cells where the voltage-gated sodium channels (Na_V_s in blue) are clustered are not labeled by either calbindin-28 kDa or calretinin. Calretinin immunolabeling is concentrated at distinct locations on the retinal ganglion cell axon (dashed red line) where they are most likely membrane bound. Calbindin-28 kDa and calretinin are either diffusely coexpressed or differentially expressed in the axons (diffuse red dots), but are both absent from the initial segments.

The major new finding reported here is that the subcellular distribution of both calretinin and calbindin-28 kDa are non-uniform in RGCs. In both cases, even in cells where they brightly stain the soma, staining is excluded from the dendritic arbors, with, in most cases, the CBP excluded even from the dendritic trunks closest to the soma. Similarly, although staining was found for both CBP on either side of the initial segments of RGC axons—that is, in the somata and nerve fibers—they were excluded from the initial segments. Thus even though the cytoplasmic staining for these CBP seems reasonably uniform, suggestive of freely diffusing soluble proteins, there are clearly mechanisms, which must involve either local binding sites or active transport, that exclude these CBP from certain regions of the cell and concentrate them in others. This phenomenon is particularly striking in the case of calretinin staining of RGC axons, which reveals punctate spots of high concentration superimposed on a diffuse background of what we presume to be cytoplasmic staining. These results have been summarized as a schematic in Figure 9.

The mechanisms by which these proteins are localized are not known, but it seems likely that their localized distributions are linked to their functions. Both proteins are often regarded as contributing to shaping neuronal responses largely by serving as Ca^2+^ buffers [24]. Such buffers can determine the kinetics of changes in local Ca^2+^ concentrations; in the case of transient fluxes of Ca^2+^ into the cytoplasm through channels in the plasma membrane or endoplasmic reticulum, buffers can govern the amplitudes of such changes, as well as their time courses. The kinetics and buffering power are determined both by the intrinsic Ca^2+^-binding properties of the CBP (numbers of sites, kinetic and equilibrium constants for Ca^2+^ binding), and by the local concentrations. Calcium binding kinetics for calretinin have not been fully elucidated [53,54] but are expected to resemble calbindin-D28k for its relatively low-affinity buffering capacity and its fast calcium-binding kinetics [55,56]. It is possible that calretinin and calbindin-28 kDa may have different binding properties to other proteins which enable it to be localized in distinct loci in the RGCs to influence local fluxes of Ca^2+^. Thus it seems likely that the RGC dendrites and axonal initial segments either require lower Ca^2+^ buffering capacity than the other regions of the cell, or that alternative CBPs serve to replace calretinin and calbindin-28 kDa in these regions. Clearly, the same must be true of those RGCs that do not contain either of these proteins in detectable amounts. It is important to note here that other calcium-binding proteins, such as parvalbumin [57], are also known to be expressed in rat RGC’s but these were not tested in this study.

In addition to simply buffering Ca^2+^, CBPs can be involved in direct regulation of signaling pathways by binding to and modifying the activities of cellular proteins in a Ca^2+^-dependent way. Whether calretinin and calbindin-28 kDa play such roles in RGCs is not known, but their irregular distribution can only be explained in terms of relatively high affinity binding to other proteins. It is possible that CBPs regulate these proteins, rather than simply being localized by them.
